# A Three-Headed Piriformis Muscle With Splitting of the Common Fibular Nerve

**DOI:** 10.7759/cureus.35302

**Published:** 2023-02-22

**Authors:** Ramesses A Akamefula, Arada Chaiyamoon, Samir Anadkat, Joe Iwanaga, R. Shane Tubbs

**Affiliations:** 1 Medicine, Tulane University School of Medicine, New Orleans, USA; 2 Anatomy, Faculty of Medicine, Khon Kaen University, Khon Kaen, THA; 3 Structural and Cellular Biology, Tulane University School of Medicine, New Orleans, USA; 4 Neurosurgery, Tulane University School of Medicine, New Orleans, USA; 5 Neurology, Tulane University School of Medicine, New Orleans, USA; 6 Neurosurgery, Ochsner Neuroscience Institute, New Orleans, USA; 7 Anatomical Sciences, St. George’s University, St. George’s, GRD; 8 Surgery, Tulane University School of Medicine, New Orleans, USA

**Keywords:** piriformis, sciatic nerve, variation, anatomy, lower limb

## Abstract

Although the division of the piriformis muscle by the sciatic nerve or its branches is fairly common, other anatomical variations of this muscle are relatively uncommon. Here, we present a cadaveric case found to have an atypical composition of the piriformis muscle. During the routine dissection of the right gluteal region in an adult male cadaver, an unusual finding of the piriformis muscle was observed. Three distinct heads of the muscle were identified. In addition, one of these heads split the common fibular nerve. The anatomy and relationships of this case are presented here. Any variation in neurovasculature and musculature can be relevant for diagnosing or surgically intervening in the gluteal region. The present case is apparently unique and of archival value.

## Introduction

The piriformis muscle is located in the gluteal region. It is a lateral rotator of the hip during hip extension and an abductor during hip flexion [1]. It receives innervation from the ventral rami of L5 to S3, the S1 and S2 ventral rami contributing most. It is also commonly innervated by the superior gluteal nerve [2]. It originates on the sacrotuberous ligament and anterior surface of the sacrum, courses laterally through the greater sciatic foramen, and inserts on the superomedial aspect of the greater trochanter [3-5]. Clinically, the variations of the piriformis can be related to common fibular, superior gluteal, or sciatic nerve compression, which can lead to pain disorders such as piriformis syndrome and foot drop [6,7]. Anatomical variations of the piriformis can also decrease the sensitivity of physical exam techniques used to diagnose the source of gluteal and hip pain [8]. They can be surgically relevant when approaches for total hip arthroplasty and hip arthroscopy are considered [9]. Herein, we report an unusual case of a three-headed piriformis muscle found at dissection.

## Case presentation

**Figure 1:**
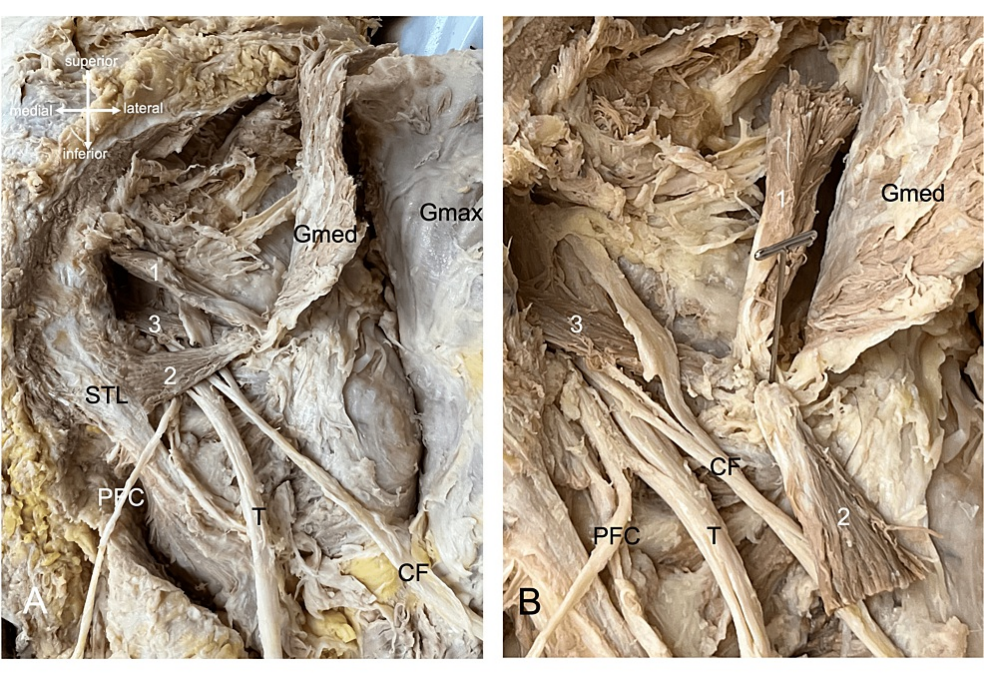
(A) Right gluteal region in the cadaveric presented in the current case. The gluteus maximus (Gmax) and gluteus medius (Gmed) muscles are reflected laterally. The superior (1), inferior (2) and middle (3) heads of the piriformis are shown. Also note the sacrotuberous ligament (STL), posterior femoral cutaneous nerve (PFC), tibial nerve (T), and common fibular nerve (CF). (B) Following reflection of the superior and inferior heads of the piriformis muscle laterally. Note the common fibular nerve is traversed by the middle head of the piriformis.

During the routine dissection (Tulane University, New Orleans, LA, USA) of the right gluteal region in a 69-year-old at-death male cadaver, an unusual finding of the piriformis muscle was observed. The piriformis was found to have three distinct heads. The superior most head arose from the edge of the greater sciatic foramen and measured 7.7 cm x 8.3 mm. The inferior head arose from the sacrotuberous ligament and measured 6.3 cm x 10 mm. The middle head arose from the anterior surface of the sacrum (S2-S4) and measured 9.4 cm x 8.6 mm. The three heads of the muscle traveled laterally to insert into the proximal femur just deep to the insertion of the gluteus medius. The superior and inferior heads of the piriformis crossed superficially to the components of the sciatic nerve (Figure 1A). The more deeply lying middle head pierced the common fibular nerve into more or less two equal parts. The middle head was superficial to the proximal parts of the tibial nerve (Figure 1B). The middle head was innervated by the first and second sacral ventral rami and no specific nerve branches were seen traveling to the superior and inferior heads. There was no typical “nerve to the piriformis.” The superior gluteal neurovascular bundle traveled superior to the superior head of piriformis and the inferior gluteal neurovascular bundle traveled inferior to the inferior head of piriformis. No other anatomical variants were observed in the gluteal region and no variants of the piriformis muscle were found on the contralateral side.

## Discussion

Anatomical variants in the relationship between the piriformis muscle and sciatic nerve have been described in the literature and these are the most common and well-known variations involving the muscle. Beaton et al. illustrated six conformations of this relationship [10]. However, they did not mention the most common variant, in which the piriformis is split by the common fibular nerve, which can ultimately lead to compression of the common fibular nerve leading to entrapment. This condition can present as an array of sensory and motor impairments. The common fibular nerve innervates the anterolateral portion of the leg and dorsum of the foot, and entrapment by the piriformis can present as burning, tingling, numbness, and pain in these areas. Impaired motor functioning of this nerve can limit individuals’ ability both to evert and to dorsiflex the ankle, leading to foot drop [6,11]. The six conformations reported by Beaton et al. are: standard textbook anatomy, a split sciatic nerve passes both through and below the piriformis muscle, a split sciatic nerve passes both above and below the piriformis muscle, the sciatic nerve splits the piriformis muscle belly, a split sciatic nerve passes both through and above the piriformis muscle, and the sciatic nerve travels superior to the piriformis muscle belly. Knowledge and recognition of these variations can be crucial for establishing a differential diagnosis. Cadaveric studies have revealed anatomical variants of the piriformis muscle and sciatic nerve in 16.9% of specimens [12]. Magnetic resonance imaging has revealed variations in the relationship between the sciatic nerve and piriformis muscle in 19.2% of patients [13]. Nevertheless, both Smoll et al. [12] and Bartret et al. [13] found no difference in the incidence of piriformis syndrome between patients with traditional anatomy and those with one of the variations, though some variations can lead to impingement of the sciatic nerve, leading to sciatica and pain in the gluteal region. Referred gluteal pain and signs of sciatica due to sciatic nerve entrapment can entail a decrease in sensitivity in physical examination maneuvers and techniques that rely on a passive range of motion, emphasizing the need for a strong understanding of the piriformis and its variants for reaching a differential diagnosis when a patient with pain in the gluteal region is evaluated. Other anatomical variants of the piriformis muscle include differing distances between the musculotendinous junction and its insertion onto the greater trochanter. Windisch et al. described three subtypes of this variant. In subtype A, there was a greater distance between the musculotendinous junction and insertion in the superior belly of the piriformis; in subtype B, there was a greater distance between the musculotendinous junction and insertion in the inferior belly; and in subtype C, the distance between the musculotendinous junction and insertion was the same in both bellies of the muscle. Windisch et al. also found variations in which the piriformis tendon fused with adjacent tendons including the superior gemellus, obturator internus, and gluteus medius tendons [14]. These anatomical variations might contribute to entrapment and irritation of articulating nerves such as the sciatic nerve, possibly leading to conditions such as piriformis syndrome and sciatica. Moreover, inflammation of the piriformis under these variant conditions can lead to myositis ossificans and myofascial syndrome, illustrating the importance of understanding the different variants of the muscle [15,16]. The absence of the piriformis muscle has been described by authors such as Brenner et al. and Leal et al. [17,18]. Lastly, the piriformis has been reported to have rarely, two heads with possible attachments to the sacrotuberous ligament, sacrum, or ilium, and recently, in a single case report, three heads but without splitting of the sciatic nerve such as was identified in the case presented herein [19]. The size of the three heads in our case compared to that of Kozioł et al. [19] was comparable although the middle head in our case was shorter. Additionally, Deopujari et al. reported a three-headed piriformis found in a cohort of 21 cadavers [20]. However, this case from these authors had a different branching pattern of the common fibular and tibial nerve compared to ours although the innervation of the piriformis was similar. The dorsal muscle mass of the lower limb gives rise to the muscles of the gluteal region. Therefore, the derailment of embryological processes in this region could lead to the maldevelopment of regional muscles such as the piriformis.

## Conclusions

We believe that familiarity with the piriformis and surrounding muscles in the gluteal region is clinically significant not only in respect of piriformis syndrome, but also in the application of hip arthroplasty approaches, hip trauma, imaging interpretation, and surgical intervention. The clinical presentation of pain syndromes due to anatomical variants of the piriformis can be similar to back pain associated with L5/S1. These similarities in clinical presentations further illustrate the need for a strong understanding of the different anatomical variants of the piriformis for reaching a differential diagnosis. The case presented herein appears to be a previously unreported variant of the piriformis muscle and is of archival value.
